# Silencing of SENP2 in Multiple Myeloma Induces Bortezomib Resistance by Activating NF-κB Through the Modulation of IκBα Sumoylation

**DOI:** 10.1038/s41598-020-57698-0

**Published:** 2020-01-21

**Authors:** Hongyi Xie, Yuanliang Gu, Wenjuan Wang, Xuyao Wang, Xiaojuan Ye, Chao Xin, Mengjiao Lu, B. Ashok Reddy, Peng Shu

**Affiliations:** 1 Clinical Laboratory, the People’s Hospital of Beilun District, Beilun Branch Hospital of the First Affiliated Hospital of Medical School Zhejiang University, 1288 Lushan East Road, Beilun District Ningbo, 315800 China; 2 Department of prevention and health care, the People’s Hospital of Beilun District, Beilun Branch Hospital of The First Affiliated Hospital of Medical School Zhejiang University, 1288 Lushan East Road, Beilun District Ningbo, 315800 China; 3 Department of Hematology & Oncology, the People’s Hospital of Beilun District, Beilun Branch Hospital of the First Affiliated Hospital of Medical School of Zhejiang University, 1288 Lushan East Road, Beilun District Ningbo, 315800 China; 4 Molecular Laboratory, the People’s Hospital of Beilun District, Beilun Branch Hospital of The First Affiliated Hospital of Medical School Zhejiang University, 1288 Lushan East Road, Beilun District Ningbo, 315800 China; 5 Division of Oncology, Liveon Biolabs, 46 & 47, Water tank Road, KIADB-Phase II, Antharasanahalli, Tumakuru Karnataka, PIN-572106 India

**Keywords:** Proteases, Tumour-suppressor proteins

## Abstract

The proteasome inhibitor bortezomib is the most successfully applied chemotherapeutic drug for treating multiple myeloma. However, its clinical efficacy reduced due to resistance development. The underlying molecular mechanisms of bortezomib resistance are poorly understood. In this study, by combining in silico analysis and sgRNA library based drug resistance screening assay, we identified SENP2 (*Sentrin/SUMO-specific proteases-2*) as a bortezomib sensitive gene and found its expression highly downregulated in bortezomib resistant multiple myeloma patient’s samples. Furthermore, down regulation of SENP2 in multiple myeloma cell line RPMI8226 alleviated bortezomib induced cell proliferation inhibition and apoptosis, whereas, overexpression of SENP2 sensitized these cells to bortezomib treatment. We further demonstrate that knockdown of SENP2 in RPMI8226 cells increased SUMO2 conjugated IκBα that resulted in the activation of NF-κB. Taken together, we report that silencing of SENP2 and consequent activation of NF-κB through the modulation of IκBα sumoylation as a novel mechanism inducing bortezomib resistance in multiple myeloma.

## Introduction

Multiple myeloma (MM) is a malignant plasma cell tumor that account for 12% of blood malignant tumors, which is the second most commonly diagnosed hematologic malignancy^1^. Despite applying multiple therapeutic drugs such as, proteasome inhibitor bortezomib^2^ and immunomodulator thalidomide^3^, the median MM patient survival was only slightly improved from 3–5 years to 5–7 years^4^. Moreover, MM is still featured as a recurrence and insensitive to treatment due to drug resistance development^5^.

The bortezomib is the first class of proteasome inhibitor approved for the treatment of MM. Bortezomib is a dipeptidyl boronic acid that reversibly binds to chymotrypsin-like catalytic subunit of 20 S proteasome and inhibits its activity^6^. Proteasome plays a critical role in cellular homeostasis by degrading unwanted cellular proteins^7^. The imbalance between synthesis and degradation of proteins arises due to proteasome inhibition results in the accumulation of regulatory proteins causing endoplasmic reticulum stress and activation of the unfolded protein response leading to induction of apoptosis in malignant cells via multiple mechanisms^8,9^.

In preclinical studies bortezomib showed anti-multiple myeloma effects, such as, inhibition of cell growth promoting ΝF−κΒ (*Nuclear factor kappa-light-chain-enhancer of activated B cells*) pathway^10^, induction of apoptosis by tumor suppressor p53^11^, inhibition of cell cycle and induction of apoptosis by Bcl2^12^. Bortezomib treatment alone or in combination with dexamethasone significantly increased the overall survival and relapse free survival in MM patients^13^. Furthermore, bortezomib shown synergistic effect when treated in combination with dexamethasone and immunomodulator, the response rates were 30%, and 60–90%, respectively^14^. Despite initial clinical success, MM patients eventually developed resistance to bortezomib therapy and thus limiting its clinical efficacy^15,16^.

Sumoylation is a process of posttranslational modification of target proteins by small ubiquitin-like modifier (SUMO). The attachment of SUMOs is catalyzed by an enzymatic cascade consists of an activating enzyme (E1), a conjugating enzyme (E2) and a ligase (E3). Sumoylation is a reversible process; SUMO detachment is carried out by SUMO proteases, originally called as Sentrin-specific Proteases (SENPs), which detaches SUMOs from the target proteins through a process called deSUMOylation^17^. Sumoylation plays a crucial role in various cellular processes such as gene regulation, genome stability, DNA damage response, protein trafficking, signal transduction and cell cycle control^18–20^. Due to its involvement in various cellular processes, not surprisingly dysregulation of sumoylation pathway is implicated in tumorigenesis and drug resistance in many cancers including MM^21,22^.

In this study, we combined in silico analysis and bortezomib drug resistant screening assay to identify the genes, which contribute to the development of bortezomib resistance in MM. We identified SUMO-specific protease, SENP2 as a significantly downregulated gene in bortezomib resistant MM patient’s samples and found it as a sensitive gene in bortezomib resistance screening assay. Furthermore, we show that loss of SENP2 expression potentiates bortezomib resistance development by activating NF-κB through the modulation of IκBα sumoylation.

## Results

### SENP2 is a bortezomib sensitive gene and its expression is highly downregulated in bortezomib resistant MM patient’s samples

**Figure 1:**
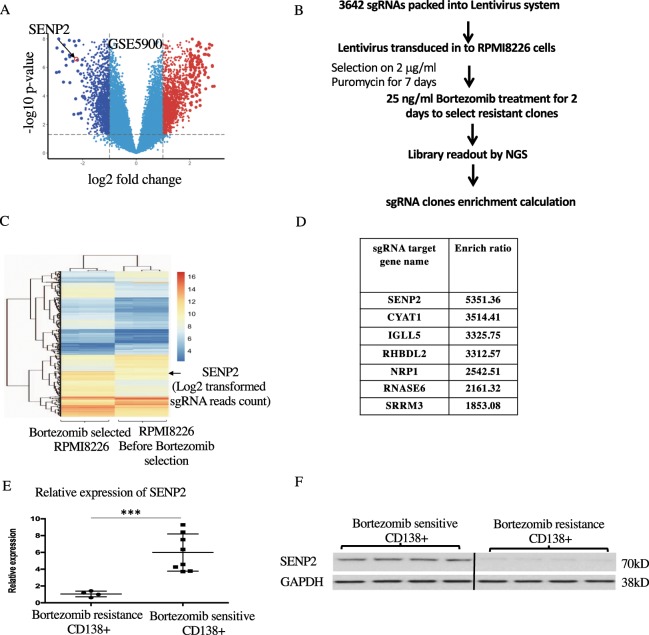
Expression of SENP2 is downregulated in bortezomib resistant MM patients: (**A**) Differential gene expression analysis using GSE5900 dataset identified 1214 down-regulated genes in MM patient samples (n = 12) compared to healthy controls (n = 22). SENP2 is indicated as a highly downregulated gene with in MM patients compared to healthy donors. (**B**) Schematic representation of sgRNA based bortezomib resistance screening assay. (**C**) The Heat maps representing next generation sequencing readouts of 126 bortezomib resistant sgRNA library clones before and after bortezomib treatment. SENP2 enrichment is indicated, which had the highest enrichment ratio among the 126 bortezomib resistant sgRNA library clones. (**D**) The next generation sequencing enrichment scores for the selected top seven sgRNA clones identified in bortezomib resistance screening assay. Among them, SENP2-sgRNA clones were found to have highest enrichment score. (**E**) RNA expression analysis of SENP2 from MM patient’s CD138 + bone marrow cells, bortezomib resistance (n = 4) and bortezomib sensitive (n = 8). (**F**) Western blot analysis to detect SENP2 protein expression levels in bortezomib resistance (n = 4) and bortezomib sensitive (n = 4) MM patient’s CD138 + bone marrow cells. Here, GAPDH serves as a loading control.

To uncover the molecular mechanisms contributing to the development of bortezomib resistance in MM, we aimed at identifying the genes whose loss of expression in MM may lead to bortezomib resistance development. For this purpose, we performed differential gene expression analysis in bone marrow plasma cells of MM patients (n = 12) and healthy donors (n = 22) from GSE5900 public dataset and found 1214 genes as significantly down regulated in MM patients compared to healthy donors. Among the down regulated genes, we found specifically SENP2 expression was highly down regulated in MM patients compared to healthy donors (Fig. 1A). However, expression of other six human SENPs (SENP1, SENP3, SENP5, SNP6, SENP7 and SENP8) were found not differentially altered in MM patients compared to healthy donors (Supplementary Fig. 1). To further validate these in silico findings, we developed sgRNA (small guide RNA) library based bortezomib resistance screening assay. For this purpose, we constructed sgRNA library for all 1214 down regulated genes in MM, for each gene 3 sgRNAs were designed, in total 3642 sgRNAs were designed. The screening was performed by using sgRNAs packed in lentivirus transduced into RMPI8226 cells as represented schematically in Fig. 1B and as described in detail in materials and methods section. With this screening and followed by next generation sequencing analysis, we identified 126 genes-sgRNA clones out of 1214 down regulated genes as highly resistant to bortezomib treatment (Fig. 1C). Among these 126 bortezomib resistant clones identified by NGS, we found SENP2-sgRNA clones had the highest enrichment ratio indicating these clones develop highest resistance to bortezomib treatment (Fig. 1D). Furthermore, we validated these in silico and *in vitro* findings by performing expression analysis of SENP2 from in-house MM clinical samples and found that SENP2 expression indeed significantly downregulated in CD138+ bone marrow cells of bortezomib resistant MM patient’s compared to bortezomib sensitive MM patient’s both at the RNA expression (Fig. 1E) and protein levels (Fig. 1F). Altogether, these results demonstrate that SENP2 as a bortezomib sensitive gene and its expression is highly down regulated in bortezomib resistant MM patients, indicating crucial role for SENP2 in bortezomib resistance development.

### SENP2 loss of expression reduced bortezomib induced cell proliferation inhibition and apoptosis in RPMI8226 cells

**Figure 2:**
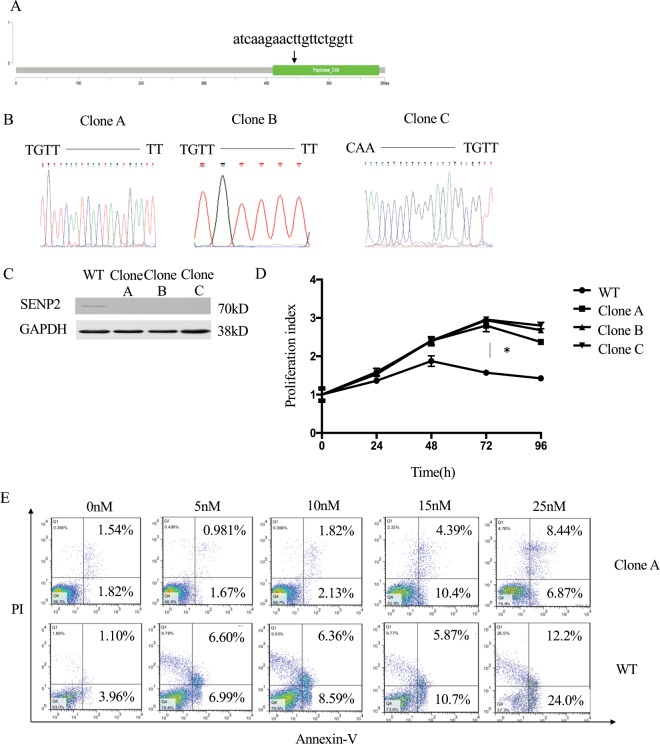
SENP2 down regulation potentiates bortezomib resistance: (**A**) Schematic view of SENP2 gene sgRNA sequence designed to target its peptidase_C48 domain. (**B**) DNA chromatograms obtained from Sanger sequencing of SENP2-sgRNA clones, wherein, 3 types of mutations are inserted in peptidase_C48 domain, which are named as Clone A, B, and C. (**C**) Western blot analysis to detect SENP2 protein expression in SENP2-sgRNA clones A, B, and C, compared to wild type (WT) cells. Here, GAPDH serves as a loading control. (**D**) SENP2 wild type (WT) cells and SENP2-sgRNA Clone A, B, C cells were treated with 25 nM bortezomib and cell proliferation at different time points were analysed by using CCK8 kit. (E) SENP2 wild type (WT) cells and SENP2-sgRNA clone-A cells were treated with 0, 5, 10, 15, 25 nM of bortezomib for 48 hours. Then, apoptosis was measured by Annexin-V APC/PI double staining.

Bortezomib exerts antitumor activities in MM through the inhibition of cell proliferation and induction of apoptosis. Therefore, we reasoned that SENP2 loss of expression could adversely affect the bortezomib induced cell cycle proliferation inhibition and apoptosis resulting in resistance development. To investigate this, we extracted SENP2-sgRNA clones from library, which have shown highest enrichment ratio in bortezomib resistance screening assay, wherein, SENP2 loss of expression was achieved by targeting its catalytic peptidase_C48 domain (Fig. 2A). As described earlier, SENP2-sgRNAs were packaged into lentivirus and transduced into RPMI8226 cells. After the selection of sgRNA clones, the insertion of mutations in peptidase C48 domain of SENP2 were confirmed by sequencing analysis (Fig. 2B). As expected all of these 3 SENP2-sgRNA clones (Clone-A, Clone-B and Clone-C) shown complete loss of SENP2 protein expression compared to wild type cells (Fig. 2C). Furthermore, all these three SENP2-sgRNA clones showed significant reduction in bortezomib induced cell proliferation inhibition (Fig. 2D). Additionally, Clone-A cells were found to suppress induction of apoptosis upon bortezomib treatment (Fig. 2E). Similarly, Clone-B and Clone-C cells also shown to reduce the bortezomib induce apoptosis (Supplementary Fig. 2). Taken together, these results demonstrate that SENP2 as a critical mediator for bortezomib induced anti-cell proliferation and apoptosis; thus, its loss of expression potentiates resistance development to bortezomib treatment in RPMI8226 cells.

### Exogenous expression of SENP2 specifically abrogates bortezomib resistance

**Figure 3:**
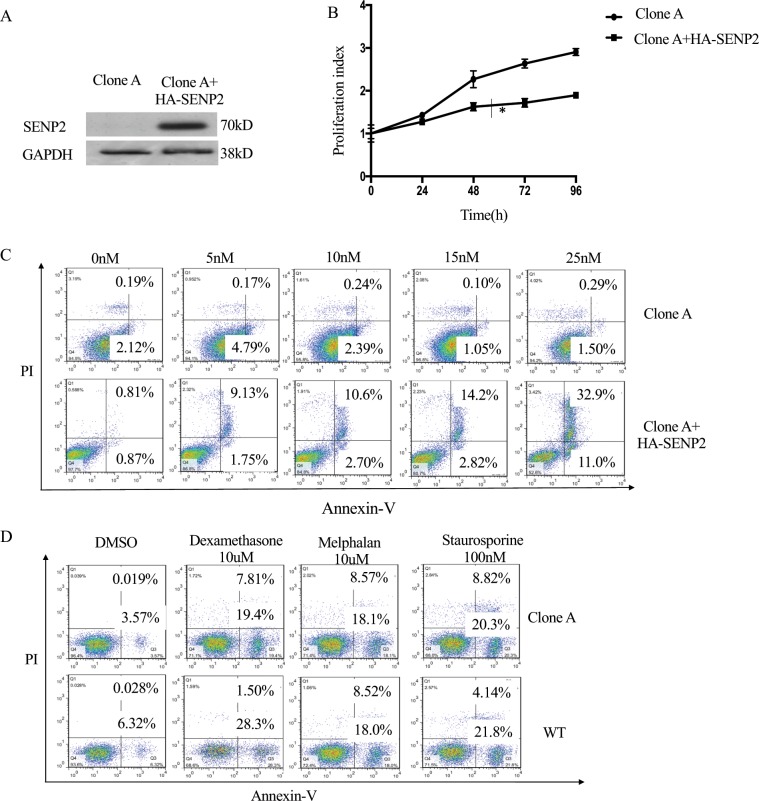
Exogenous expression of SENP2 specifically abrogates bortezomib resistance: (**A**) Exogenous expression of HA-SENP2 in Clone-A cells. After 48 hours, cells were lysed and western blot analysis was performed to detect HA-SENP2 expression. Here, GAPDH serves as a loading control. (**B**) Clone-A cells transiently transfected with control vector or HA-SENP2 were treated with 25 nM bortezomib for 48 hours. Then, cell proliferation was analysed by using CCK8 kit. (**C**) Clone-A cells transiently transfected with control vector or HA-SENP2 were treated with 0, 5, 10, 15, 25 nM of bortezomib for 48 hours and then apoptosis was measured by Annexin-V and APC/PI double staining. (**D**) SENP2 wild type cells and Clone-A cells were treated with 10uM Dexamethasone, 10uM Melphalan and 100 nM Staurosporine for 48 hours, then apoptosis was analysed by Annexin-V and APC/PI double staining.

To investigate further the role of SENP2 in bortezomib resistance development, we generated HA-SENP2 overexpression vector for the exogenous expression of SENP2 in bortezomib resistant SENP2 Clone-A cells and its exogenous expression was confirmed by western blotting analysis (Fig. 3A). Moreover, exogenous expression of HA-SENP2 modified bortezomib resistance by inhibiting the cell proliferation in Clone-A cells compared to vector alone transfected cells (Fig. 3B). In addition, exogenous expression of HA-SENP2 potentiated the dose dependent apoptosis induction upon bortezomib treatment in Clone-A cells compared to vector alone transfected cells, thus abrogating bortezomib resistance in these cells (Fig. 3C). Interestingly, exogenous expression of SENP2 had no effect on induction of apoptosis upon treatment of other therapeutic drugs such as, Dexamethasone, Melphalan and Staurosporine in bortezomib resistant Clone-A cells as well as in the wild type cells, indicating the specificity of SENP2 expression in potentiating bortezomib induced apoptosis (Fig. 3D). Altogether, these results demonstrate the specific role for SENP2 in overcoming bortezomib resistance in RPMI8226 cells.

### SENP2 inhibits NF-κB activation by regulating sumoylation of IκBα

**Figure 4:**
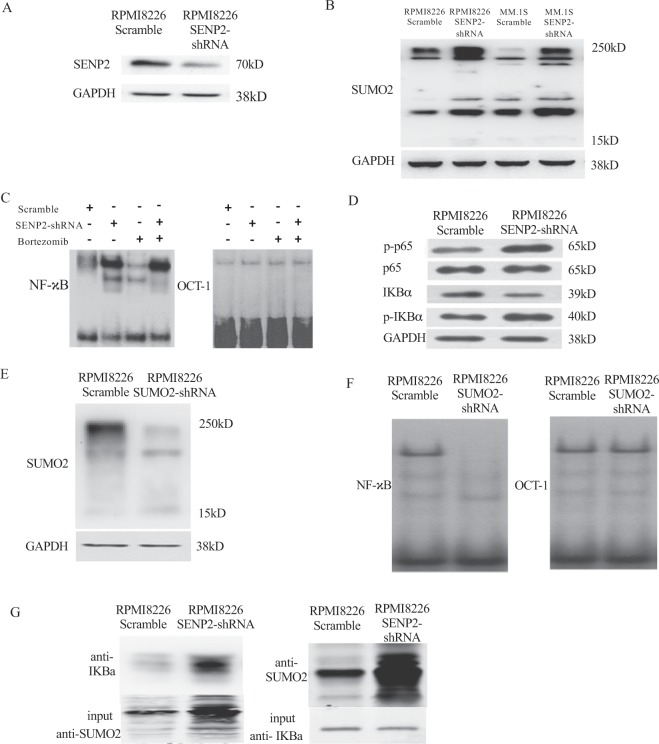
SENP2 negatively regulates NF-κB activation through the modulation of IκBα sumoylation. (**A**) Either Scramble shRNA or SENP2-shRNA were transiently transfected in RPMI8226 cells. After 48 hours, western blot analysis was performed to detect SENP2 protein levels. Here, GAPDH serves as a loading control. (**B**) MM cell lines, RPMI8226 and MM.1S were transfected with either scramble shRNA or SENP2-shRNA for 48 hours, then western blot analysis was performed to detect SUMO2 protein levels. Here, GAPDH serves as a loading control. (**C**) EMSA assay was performed to detect NF-κB binding to its consensus binding sites on biotin labelled DNA probe using cell lysates of RPMI8226 cells transfected with either Scramble shRNA or SENP2-shRNA for 48 hours and either untreated or treated with bortezomib 25 nM. Here, OCT1 serves as an internal control for EMSA assay. (**D**) NF-κB activation was analysed by western blotting analysis using cell lysates of RPMI8226 cells transfected with either scramble shRNA or SENP2-shRNA for 48 hours and followed by probing with antibodies against p-p65, p65, p-IκBα and IκBα. Here, GAPDH serves as a loading control. (**E**) Either scramble shRNA or SUMO2-shRNA were transiently transfected in RPMI8226 cells. After 48 hours, western blot analysis was performed to detect SUMO2 protein levels. Here, GAPDH serves as a loading control. (**F**) EMSA assay was performed to detect NF-κB binding to its consensus binding sites on biotin labelled DNA probe using cell lysate of RPMI8226 cell lysates transfected with either Scramble shRNA or SUMO2-shRNA for 48 hours. Here, OCT1 serves as an internal control for EMSA assay. (**G**) Reciprocal immunoprecipitation analysis using anti-IκBα and anti-SUMO2 antibodies from cell extracts of RPMI8226 cells transfected with Scramble shRNA or SENP2-shRNA for 48 hours.

SENP2 is a SUMO specific protease, which detaches SUMO modification on proteins^23^. Therefore, SENP2 may exert its anti-bortezomib resistance activity by suppressing drug resistance inducing NF-κB activity^24^ through the regulation of sumoylation of proteins that are controlling NF-κB activity. As reported by others, increase in SUMO1 and SUMO2/3 levels affects NF-κB activity. SUMO1 and SUMO2/3 have antagonistic effects on NF-κB activation through regulation of NF-κB inhibitor, IκBα (*Inhibitor of kappa B alpha*) stability. SUMO1 modification of IκBα increases its stability by inhibiting ubiquitilation-mediated degradation and consequently inhibiting NF-κB activation^25^, where as, SUMO2/3 modification of IκBα results in its degradation through ubiquitilation and thus inducing NF-κB activation^26^. Therefore, we reasoned that SENP2 regulation of NF-κB activation is mediated by SUMO2 in inducing bortezomib resistance. To investigate this, we knockdown SENP2 using shRNA in RPMI8226 cells (Fig. 4A), as expected increase in SUMO2 protein levels was observed in SENP2 knockdown condition as compared to control knockdown condition (Fig. 4B). We further confirm the increase in SUMO2 protein levels upon SENP2 knockdown in another MM cell line, MM.1 S (Fig. 4B). Whereas, SUMO1 levels are not affected by SENP2 knockdown in RPMI8226 and MM.1 S Multiple myeloma cell lines, indicating specific role for SENP2 in regulating SUMO2 levels in these cells (Supplementary Fig. 3). Furthermore, we knockdown SENP2 and found increased NF-κB activation upon bortezomib treatment by electrophoretic mobility shift assay (EMSA) as assessed by increase in NF-κB binding to its consensus sites on biotin labeled DNA probe compared to control condition, herein, OCT1 serves as a internal EMSA control (Fig. 4C). Moreover, increase in the phosphorylation of p65 and IκBα were observed in SENP2 knockdown cells, which are the known markers of NF-κB activation (Fig. 4D), suggesting SENP2 as a negative regulator of NF-κB activation. In contrast, knockdown of SUMO2 (Fig. 4E) resulted in decrease in NF-κB binding to its consensus binding sites on biotin labeled DNA probe, suggesting SUMO2 as crucial mediator in SENP2 mediated inhibition of NF-κB activation (Fig. 4F). Then, we investigated whether SUMO2 modification of IκBα is essential for SENP2 mediated NF-κB regulation. For this purpose, we knockdown SENP2 and found increase in SUMO2 conjugated IκBα by immunoprecipitation analysis (Fig. 4G), and decrease in IκBα protein levels by western blot analysis (Fig. 4D), suggesting SENP2 controlled activation of NF-κB is via regulation of IκBα sumoylation and its degradation. Altogether these results demonstrate that SENP2 negatively regulates NF-κB activation through the modulation of IκBα sumoylation.

## Discussion

Despite initial clinical success in treating MM, many patients eventually relapsed from bortezomib therapy due to resistance development^27,28^. Bortezomib resistance is caused due to mutations in the binding pocket of PSMB5 subunit of proteasome that resulted in impaired drug binding. Additionally, upregulation of proteasomal subunits and subsequent alteration in the ratio of proteasome subunits also contributed to resistance development^29,30^. With the relevance of PSMB5 mutations in relapse of MM patients remaining elusive^28^, discovering alternate mechanisms potentiating bortezomib resistance are crucial for designing novel therapeutic interventions to treat these malignancies. Recently, unfolded protein response pathway and vesicular exocytosis of ubiquitilated proteins were reported to induce bortezomib resistance^8,31^. Besides this, constitutive activation of NF-κB is implicated in the development of bortezomib resistance in MM patients^24^. In this study, we found silencing of SENP2 in MM patients as a mechanism contributing to the development of bortezomib resistance through the activation of NF-κB. Similarly, activation of NF-κB was found to induce drug resistance in others cancers too^32^. Thus, targeting NF-κB activation is crucial for overcoming bortezomib resistance in MM and drug resistance in other cancers^33,34^.

Several components of sumoylation pathway were found dysregulated in cancers, which caused severe defects in cell proliferation and genome stability resulting in the development and progression of cancers^21,22^. Similarly, dysregulation of sumoylation pathway was found to be associated with adverse patient outcome in MM. The SUMO conjugating enzyme UBE2I and SUMO-ligase PIAS1 were found to be upregulated in MM patients, which correlated with the poor patient survival^21^. Additionally, SUMO-specific protease SENP1 was found aberrantly expressed in MM cells, which is associated with enhanced cell proliferation^35^. However, role of sumoylation in drug resistance development in MM is not well understood. In this study, we found SUMO-specific protease SENP2 expression anti-correlated with bortezomib resistance in MM patients. Furthermore, we found SENP2 modulation of bortezomib resistance is through inhibiting NF-κB activity via regulation of IκBα sumoylation. In agreement with our findings, Gao *et al*.^36^ found that SENP2 sensitizing breast cancer cells to doxorubicin drug resistance through the inhibition of NF-κB activity^36^. Thus, SENP2 plays a critical role in overcoming drug resistance in cancers.

**Figure 5:**
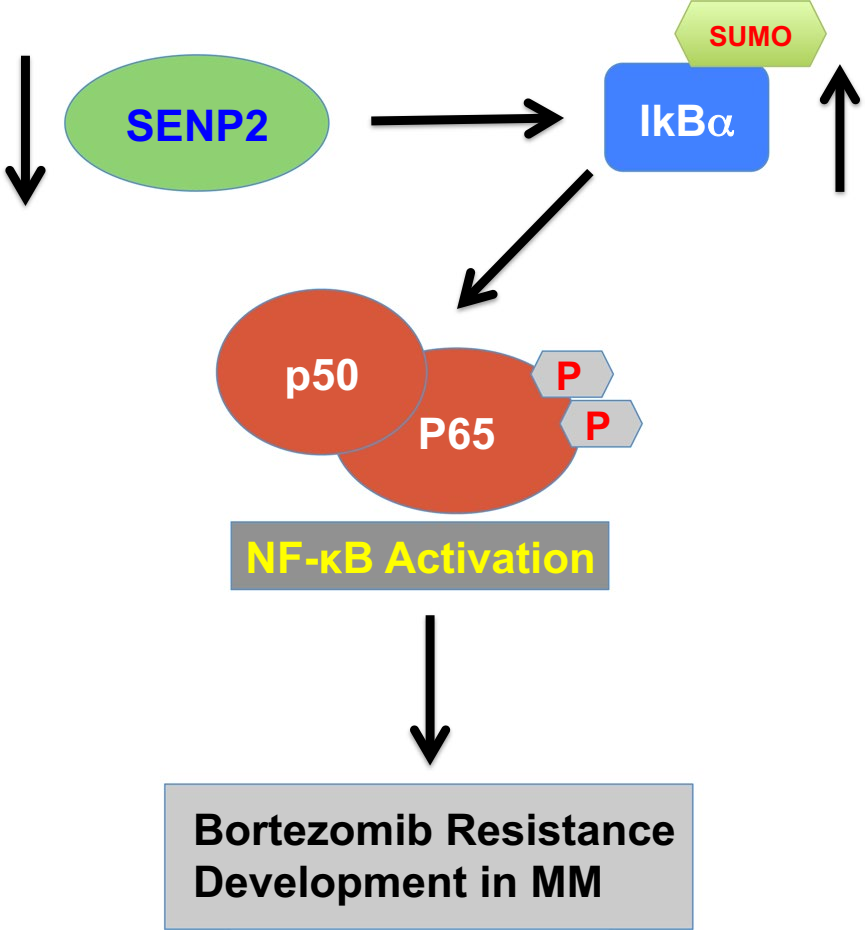
A schematic model depicting SENP2 role in bortezomib resistance development in MM: SENP2 loss of expression results in increased IkBa sumoylation that consequently leads to the activation of NF-κB, which potentiates bortezomib resistance development in MM.

SENP2 is a tumor suppressor; its expression is silenced in osteosarcoma^37^. Similarly, we found SENP2 expression silenced in bortezomib resistant MM. While the mechanisms controlling SENP2 expression in cancers remain unknown, NF-κB shown to induce the expression of SENP2 under genotoxic stress condition. Thus, there is a negative feedback mechanism, wherein, NF-κB activates SENP2 expression and inturn SENP2 inhibits NF-κB activity^38^. Despite constitutive expression of NF-κB in cancers, SENP2 expression is found silenced, indicating additional epigenetic mechanisms operating in silencing of SENP2 expression, which could therefore serve as attractive targets for treating bortezomib resistant MM. Our findings suggest that silencing of SENP2 leads to activation of NF-κB through the modulation of IκBα sumoylation resulting in bortezomib resistance development in MM. Thus, identifying SUMO-ligases that target IκBα could also serve as additional novel targets for treating bortezomib resistant MM. However, additional research is demanded to uncover epigenetic mechanisms silencing SENP2 expression and also to identify aberrantly expressed SUMO-ligases of IκBα in MM. In summary, loss of SENP2 expression in MM leads to increase in IκBα sumoylation that results in the activation of NF-κB, which induces bortezomib resistance in MM (Fig. 5).

## Methods

### Ethics statement

All the multiple myeloma patients have signed informed consent forms. The present experiments including human and animal subjects, operating procedures and protocols were approved by the Ethics Committee of Ningbo Beilun People’s Hospital. All the experiments in this study were performed in accordance with the relevant guidelines and regulations formulated by the Ningbo Beilun People’s Hospital, Ningbo city, Zhejiang province, China and by following international guidelines.

### In silico data analysis

All the in silico analysis was performed by using R 3.4.3. To narrow down bortezomib sensitive genes, we first extracted GSE5900 gene expression dataset from GEO (Gene Expression Omnibus) database, healthy donors (n = 22), MGUS (Monoclonal Gammopathy of Unknown Significance, n = 44) and smoldering MM (n = 12) patients were present in this dataset. The following criteria’s are followed for the analysis; (1) Gene expression value was log2 transformed, standardized by preprocess Core package. (2) Using healthy donor (n = 22) as control, differential expressed genes in MM patients (n = 12) were calculated by limma package, FC < −1 and p < 0.05 (FDR adjusted) were identified as down-regulated genes.

### sgRNA library preparation and bortezomib resistance screening assay

The steps followed for the sgRNA library preparation and sequencing are as follows. (1) sgRNA library design, synthesis and amplification were performed by Yuwenbo biological company (Shanghai, China). sgRNA library backbone selected was Lenti Crispr V2 vector and 3 sgRNAs were designed for each gene. (2) sgRNA lentivirus library is prepared by using 2^nd^ generation of lentivirus package system. The lentivirus package vector PMD2.G (12259) and pSPAX2 (12260) were purchased from Addgene (USA). In our test system, each 10-cm^2^ dish would generate 5 million library virus particles. For this purpose, we transfected 3 μg PMD2.G, 9 μg pSPAX2 and 12 μg of sgRNA library plasmids in 293 T cells to achieve the library coverage reached 1000. Then the cell culture supernatant containing lentivirus was collected and lentivirus were concentrated by using ultracentrifugation. (3) To achieve 0.1 Multiplicity of infection (MOI, virus:cell = 1:10), we infected 50 million RPMI8226 cells with 5 million lentivirus particles in 15-cm2 dish, after 48 h, 2 ug/ml puromycin was added and selected for another 7 days to select only lentivirus infected cells. Then the RPMI8226 cells were cultured in complete medium for 48 h. (4) Further, RPMI8226-sgRNA library cells were selected by treating 25 nM bortezomib for 2 days for bortezomib resistance screening. Before selection, 2 million library cells were saved, which served as a control. (5) After bortezomib selection, genome DNA were extracted from control and sgRNA library cells and amplified by Phusion Flash High Fidelity Master Mix (Thermo), primer sequence was F1 5′-CCCGAGGGGACCCAGAGAG-3′, R1 5′-GCGCACCGTGGGCTTGTAC-3′, then barcode and index was added by second amplification. (6) Then, the libraries were sequenced by NGS (Next-Generation Sequencing). NGS measurement was performed by BGI company (Shenzhen, China). The library mapping and visualization was performed by MAGeCK-VISPR program (X. Shirley Liu Lab, https://bitbucket.org/liulab/mageck-vispr/).

### Patient sample CD138+ cell separation

Patients’ bone marrow mononuclear cells were first separated by Ficoll-Hypaque (SigmaeAldrich, St Louis, MO, USA). Then, the CD138+ cells were sorted by using EasySep™ Human CD138 Positive Selection Kit II (STEM CELL, USA).

### Real time Q-PCR assay

Total RNA from patients sample was extracted by using Trizol reagent (Invitrogen, Carlsbad, CA, USA) and cDNA libraries were prepared by following standard procedures. Then Q-PCR analysis was performed to detect gene expression levels using TB Green Premix Ex Taq II(Clontech, Dalian, China) in 7500 fast system. The primer sequence sets of SENP2 used are SENP2-F, 5′CAGAGACGATGGTCGGAATCAG3′, SENP2-R, 5′CCTCCTGAGTAAGCCATTGCTTC3′. The GAPDH primers sequence used are GAPDH-F, 5′GTCTCCTCTGACTTCAACAGCG3′, GAPDH-R, 5′ACCACCCTGTTGCTGTAGCCAA3′. The relative expression level of SENP2 was determined by 2^−ΔΔCT^ value. The experiment is performed in triplicates and the average values are represented as a fold change in gene expression. The significance was determined by the T-Test and represented as p-value.

### Cell proliferation and apoptosis assays

1. Cell proliferation assay: The cells were grown to subconfluence in 96-wells plate and cell proliferation was determined by using Cell Counting Kit-8 (CCK-8) (Dojindo molecular technologies, Rockville, MD, USA).
2. Apoptosis assay: Cells were labeled with APC-conjugated Annexin-V (BD Biosciences, San Jose,CA)/propidium iodide(PI), then apoptosis was detected using FACS caliber (BD Biosciences, San Jose,CA).

### EMSA (electrophoretic mobility shift assay)

For this purpose, nuclear and cytoplasmic proteins were fractioned by using cell protein fractionation kit (Sangon Biotech, Shanghai, China). Then the nuclear fraction was applied to the DNA probes of NF-κB binding sites, 5′AGTTGAGGGGACTTTCCCAGGC3′ and internal control OCT-1 DNA probe 5′TGTCGAATGCAAATCACTAGAA3′, which were labeled with biotin using EMSA Probe Biotin Labeling Kit (Beyo- time, Nantong, JS, China). And then bound nuclear proteins were detecting by Chemiluminescent EMSA Kit (Beyotime, Nantong, JS, China).

### Endogenous immunoprecipitation (IP)

Cell lysis was prepared by using IP lysis buffer and then labeled with SUMO2 antibody. After incubated overnight, then labeled with anti-rabbit IgG-IP Beads (Sigma Aldrich, St Louis, MO, USA). After immunoprecipitation, IκBα-SUMO2 was detected by IκBα antibody by western blotting analysis.

### Statistical analysis

Significance levels in this study were determined by Student’s t test, * equals p < 0.05, ** equals p < 0.01, *** equals p < 0.001.

### Ethics approval and consent to participate

This study was conducted in accordance with the Declaration of Helsinki principles and approved by the Medical Research Ethics Committee of Zhejiang University School of Medicine First Affiliated Hospital Beilun Branch.

## Supplementary information

Supplementary information

## References

[1] Kazandjian D (2016). *Multiple myeloma epidemiology and survival: A unique malignancy*. Semin. Oncol..

[2] Scott K (2016). *Bortezomib for the treatment of multiple myeloma*. Cochrane Database Syst. Rev..

[3] Holstein SA, McCarthy PL (2017). *Immunomodulatory Drugs in Multiple Myeloma: Mechanisms of Action and Clinical Experience*. Drugs.

[4] Sonneveld P (2016). *Treatment of multiple myeloma with high-risk cytogenetics: a consensus of the International Myeloma Working Group*. Blood.

[5] Cagnetta A (2015). *Mechanisms and Clinical Applications of Genome Instability in Multiple Myeloma*. Biomed. Res. Int..

[6] Accardi F (2015). *Mechanism of Action of Bortezomib and the New Proteasome Inhibitors on Myeloma Cells and the Bone Microenvironment: Impact on Myeloma-Induced Alterations of Bone Remodeling*. Biomed. Res. Int..

[7] Chen D (2011). *Bortezomib as the first proteasome inhibitor anticancer drug: current status and future perspectives*. Curr. Cancer Drug. Targets.

[8] Obeng EA (2006). *Proteasome inhibitors induce a terminal unfolded protein response in multiple myeloma cells*. Blood.

[9] Lipchick BC, Fink EE, Nikiforov MA (2016). *Oxidative stress and proteasome inhibitors in multiple myeloma*. Pharmacol. Res..

[10] Gupta SC (2010). *Inhibiting NF-kappaB activation by small molecules as a therapeutic strategy*. Biochim. Biophys. Acta.

[11] Ooi MG (2009). *Interactions of the Hdm2/p53 and proteasome pathways may enhance the antitumor activity of bortezomib*. Clin. Cancer Res..

[12] Smith AJ (2011). *Noxa/Bcl-2 protein interactions contribute to bortezomib resistance in human lymphoid cells*. J. Biol. Chem..

[13] Jagannath S (2004). *A phase 2 study of two doses of bortezomib in relapsed or refractory myeloma*. Br. J. Haematol..

[14] Offidani M (2013). *Efficacy and tolerability of bendamustine, bortezomib and dexamethasone in patients with relapsed-refractory multiple myeloma: a phase II study*. Blood Cancer J..

[15] Lu S, Wang J (2013). *The resistance mechanisms of proteasome inhibitor bortezomib*. Biomark Res..

[16] Zaal EA (2017). *Bortezomib resistance in multiple myeloma is associated with increased serine synthesis*. Cancer Metab..

[17] Eifler K, Vertegaal ACO (2015). *SUMOylation-Mediated Regulation of Cell Cycle Progression and Cancer*. Trends Biochem. Sci..

[18] Muller S, Ledl A, Schmidt D (2004). *SUMO: a regulator of gene expression and genome integrity*. Oncogene.

[19] Jackson SP, Durocher D (2013). *Regulation of DNA damage responses by ubiquitin and SUMO*. Mol. Cell.

[20] Melchior F, Schergaut M, Pichler A (2003). *SUMO: ligases, isopeptidases and nuclear pores*. Trends Biochem. Sci..

[21] Driscoll JJ (2010). *The sumoylation pathway is dysregulated in multiple myeloma and is associated with adverse patient outcome*. Blood.

[22] Seeler JS, Dejean A (2017). *SUMO and the robustness of cancer*. Nat. Rev. Cancer.

[23] Mukhopadhyay D, Dasso M (2007). *Modification in reverse: the SUMO proteases*. Trends Biochem. Sci..

[24] Markovina S (2008). *Bortezomib-resistant nuclear factor-kappaB activity in multiple myeloma cells*. Mol. Cancer Res..

[25] Desterro JM, Rodriguez MS, Hay RT (1998). *SUMO-1 modification of IkappaBalpha inhibits NF-kappaB activation*. Mol. Cell.

[26] Aillet F (2012). *Heterologous SUMO-2/3-ubiquitin chains optimize IkappaBalpha degradation and NF-kappaB activity*. PLoS One.

[27] Wu YX, Yang JH, Saitsu H (2016). *Bortezomib-resistance is associated with increased levels of proteasome subunits and apoptosis-avoidance*. Oncotarget.

[28] Lichter DI (2012). *Sequence analysis of beta-subunit genes of the 20S proteasome in patients with relapsed multiple myeloma treated with bortezomib or dexamethasone*. Blood.

[29] Lu S (2008). *Overexpression of the PSMB5 gene contributes to bortezomib resistance in T-lymphoblastic lymphoma/leukemia cells derived from Jurkat line*. Exp. Hematol..

[30] Balsas P (2012). *Bortezomib resistance in a myeloma cell line is associated to PSMbeta5 overexpression and polyploidy*. Leuk. Res..

[31] Leung-Hagesteijn C (2013). *Xbp1s-negative tumor B cells and pre-plasmablasts mediate therapeutic proteasome inhibitor resistance in multiple myeloma*. Cancer Cell.

[32] Bentires-Alj M (2003). *NF-kappaB transcription factor induces drug resistance through MDR1 expression in cancer cells*. Oncogene.

[33] Godwin P (2013). *Targeting nuclear factor-kappa B to overcome resistance to chemotherapy*. Front. Oncol..

[34] Baldwin AS (2001). *Control of oncogenesis and cancer therapy resistance by the transcription factor NF-kappaB*. J. Clin. Invest..

[35] Xu J (2015). *SENP1 inhibition induces apoptosis and growth arrest of multiple myeloma cells through modulation of NF-kappaB signaling*. Biochem. Biophys. Res. Commun..

[36] Gao X (2019). *SENP2 suppresses NF-kappaB activation and sensitizes breast cancer cells to doxorubicin*. Eur. J. Pharmacol..

[37] Pei H (2018). *SUMO-specific protease 2 (SENP2) functions as a tumor suppressor in osteosarcoma via SOX9 degradation*. Exp. Ther. Med..

[38] Lee MH (2011). *NF-kappaB induction of the SUMO protease SENP2: A negative feedback loop to attenuate cell survival response to genotoxic stress*. Mol. Cell.

