# Supplementary material for: Silencing of SENP2 in Multiple Myeloma Induces Bortezomib Resistance by Activating NF-κB Through the Modulation of IκBα Sumoylation
Source: Sci Rep. 2020 Jan 21;10:766. doi: 10.1038/s41598-020-57698-0 (PMC6972929; doi:10.1038/s41598-020-57698-0)
Supplement: Supplementary file 1 — Supplementary information [file 41598_2020_57698_MOESM1_ESM.pdf]

Silencing of SENP2 in Multiple Myeloma Induces Bortezomib Resistance by activating NF-κB through the modulation of IκBα Sumoylation

Hongyi Xie(xiehongyi2000@126.com)1, Yuanliang Gu(gurice@yeah.net)2, Wenjuan Wang(13655748742@163.com)3, Xuyao Wang(xuyaowang22@163.com)4, Xiaojuan Ye(yexiaojuan2014@126.com)3, Chao Xin(913625589@qq.com)1, Mengjiao Lu(1576849460@qq.com)1, B. Ashok Reddy5,Peng Shu4

The first two authors contributed equally to this paper

1 Clinical Laboratory, the People's Hospital of Beilun District, Beilun Branch Hospital of The First Affiliated Hospital of Medical School Zhejiang University, 1288 Lushan East Road, Beilun District, Ningbo 315800, China

2 Department of prevention and health care, the People's Hospital of Beilun District, Beilun Branch Hospital of The First Affiliated Hospital of Medical School Zhejiang University,1288 Lushan East Road, Beilun District, Ningbo 315800, China

3 Department of Hematology & Oncology, the People's Hospital of Beilun District, Beilun Branch Hospital of the First Affiliated Hospital of Medical School of ,Zhejiang University, 1288 Lushan East Road, Beilun District, Ningbo 315800, China

4 Molecular Laboratory, the People's Hospital of Beilun District, Beilun Branch Hospital of The First Affiliated Hospital of Medical School Zhejiang University, 1288 Lushan East Road, Beilun District, Ningbo 315800, China

5 Division of Oncology, Liveon Biolabs, 46 & 47, Water tank Road, KIADB-Phase II, Antharasanahalli, Tumakuru, Karnataka, INDIA PIN-572106

*Address correspondence to:

Peng Shu, MD.

Clinical Laboratory, the People's Hospital of Beilun District, Beilun Branch Hospital of The First Affiliated Hospital of Medical School Zhejiang University, 1288 Lushan East Road, Beilun District, Ningbo 315800, China

E-mail: m17757498873@163.com

B. Ashok Reddy

Division of Oncology, Liveon Biolabs, 46 & 47, Water tank Road, KIADB-Phase II, Antharasanahalli, Tumakuru, Karnataka, INDIA PIN-572106

E-mail: ashokreddy@liveonbiolabs.com

I. Supplementary Figures:

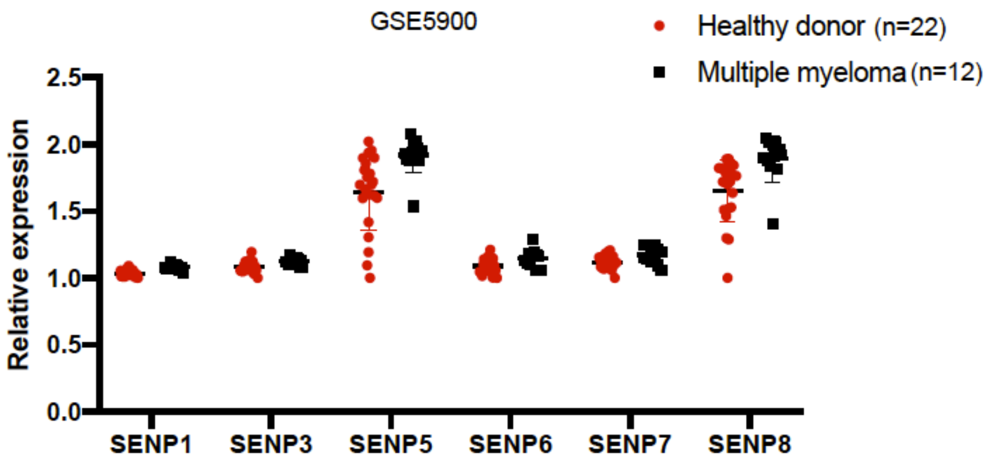

Supplementary Figure 1: Differential expression of human SENP's in GSE5900 dataset. Gene expression analysis of human SENPs, SENP1, SENP3, SENP5, SNP6, SENP7 and SENP8 using GSE5900 dataset consisting of healthy donors (n=22) and MM patient's samples (n=12).

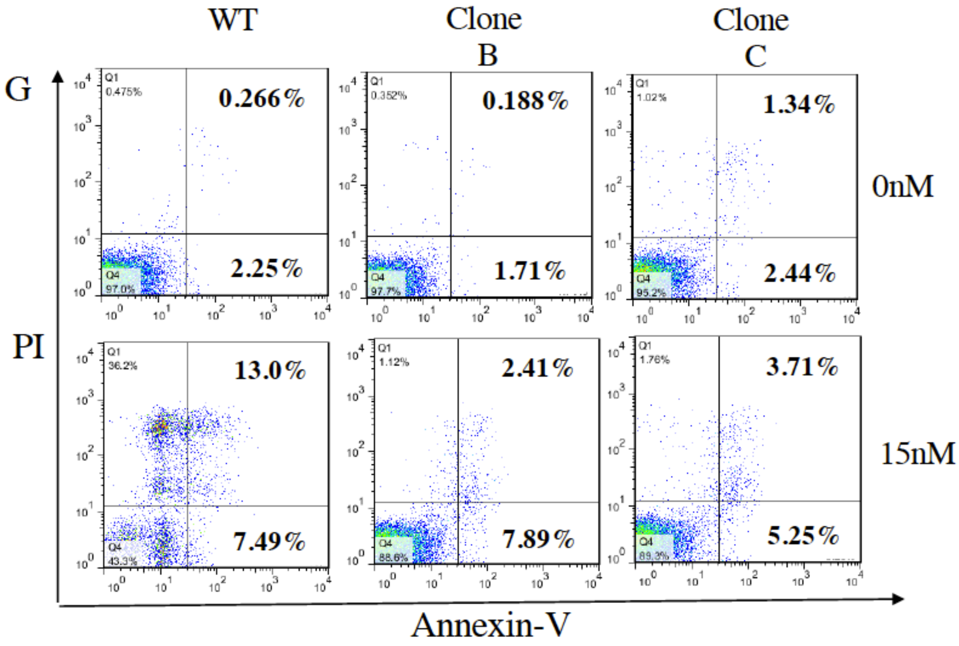

Supplementary Figure 2: SENP2 loss of expression reduces bortezomib-induced apoptosis. SENP2 wild type (WT) cells, SENP2-sgRNA Clone-B cells and Clone-C cells were either untreated or treated with 15 nM of bortezomib for 48 hours. Then, apoptosis was measured by Annexin-V and APC/PI double staining.

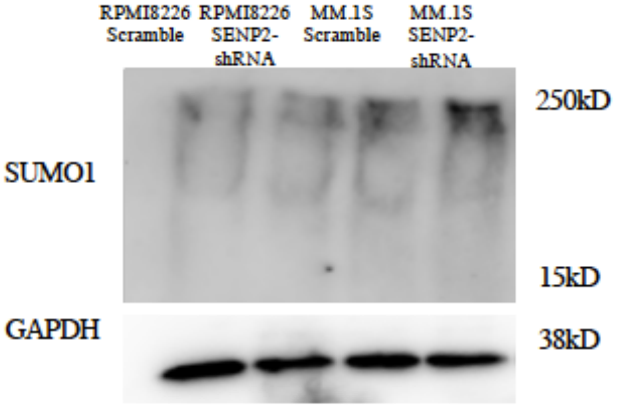

Supplementary Figure 3: SENP2 knockdown do not affect the SUMO1 protein levels. Multiple myeloma cell lines, RPMI8226 and MM.1S were either transfected with scramble shRNA or SENP2-shRNA for 48 hours, then western blot analysis was performed to detect SUMO1 protein levels. Here, GAPDH serves as a loading control.

II. Supplementary Table 1: Primer sequences of SENPs used for gene expression analysis

Name of the gene	Primer Sequence
SENP1 Forward	CATTTCGCCTGACCATTACACGC
SENP1 Reverse	CACACTTGGCAAGCCCTTCTCT
SENP3 Forward	ATCCACCTGGAGGTGCATTGGT
SENP3 Reverse	TCTTTACCGCCTCTGCCTGTAG
SENP5 Forward	GTCAGAAAGCCTCTCCAGTGGA
SENP5 Reverse	CAAGGACTTCTTTTTCACTGAGTG
SENP6 Forward	GAGCATCAAAGGAAGTTGTGGGC
SENP6 Reverse	GAAGATGGTGTGGTTTTCTCCAG
SENP7 Forward	TCTTTCCCTGCTGGTGTTGCTG
SENP7 Reverse	CAACCGCTACTTTGCTTCTGCAG
SENP8 Forward	GGCAGAAAAGGAGACAAACTGGC
SENP8 Reverse	CAGCAGTGATTCTGTCTGTTGCC

III. Supplementary Materials and Methods:

Patient's treatment and samples collection procedures

All the patients in the study group were applied VD therapy procedure and every course of treatment followed for a period of 3 weeks. During each course of treatment, bortezomib 0.7-1.3 mg/ml was injected intravenously on the days 1, 4, 8 and 11 for 2 times and dexamethasone 20-40 mg/ml intravenously on the days 1 to 4 and 9 to 12. Treatment response was assessed by EBMT (European Society for Blood and Marrow Transplantation). Among these, total of 8 patients were treated effectively (4 achieved near complete remission (nCR), 3 partial remission (PR), and 1 mild response) and therefore, they were designated as bortezomib sensitive patients. Another 4 patients shown no response to the treatment and therefore, these were designated as bortezomib resistant patients. Bone marrow was obtained for all these patients from Ningbo Beilun People's Hospital. The protocols followed for treating patients, sample collection and extraction are as per the Declaration of Helsinki and as authorized by the Institutional Ethics Board of Ningbo Beilun People's Hospital.

Cell culture procedures

Human multiple myeloma cell lines RPMI8226 and MM.1S were purchased from ATCC (The American Type Culture Collection) company(USA), culture medium is 1640+10%FBS, and culture condition is 37 °C in the presence of 5% CO_2. Sub culturing of cells were performed as per the ATCC instructions and standard cell culture practices.

Western blot analysis

Cell lysis was performed using RIPA buffer. The concentration of the total protein extracted was determined by the Lowry's method and equal quantity of protein extracts were loaded on to 12% SDS-PAGE gels and transferred on to the nitrocellulose membranes. Then, western analysis was performed by probing with primary antibodies against SENP2 (SUMO-specific proteases-2), p65, p-p65, IκBα, p-IκBα, SUMO2/3, GAPDH (Cell signaling, MA, USA), and horseradish peroxidase-conjugated secondary antibody (ZhongShan Golden Bridge Biotechnology, Beijing, China). Finally, protein expression was determined by chemiluminescence system from Pirece Biotechnology, IL, USA.

IV. Full length of gels figure:

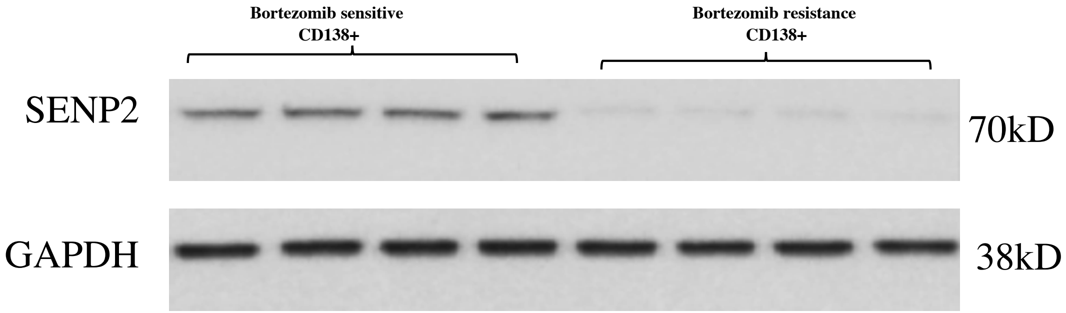

Supplementary Figure 4: Full length western blot of Figure 1 G

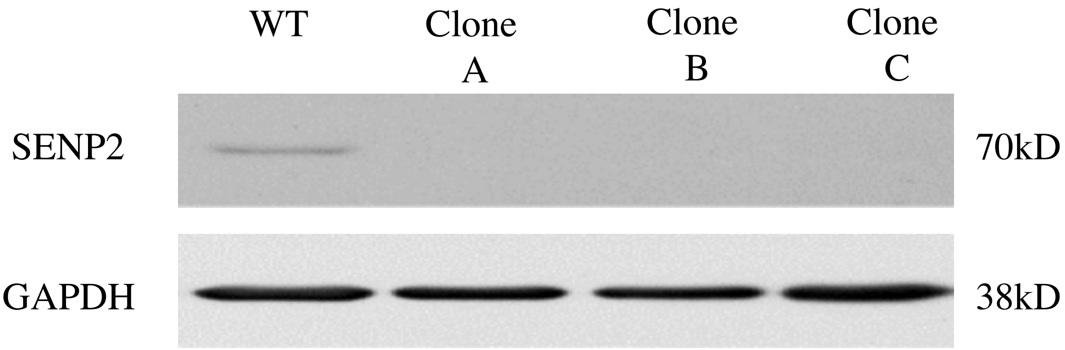

Supplementary Figure 5: Full length western blot of Figure 2 C

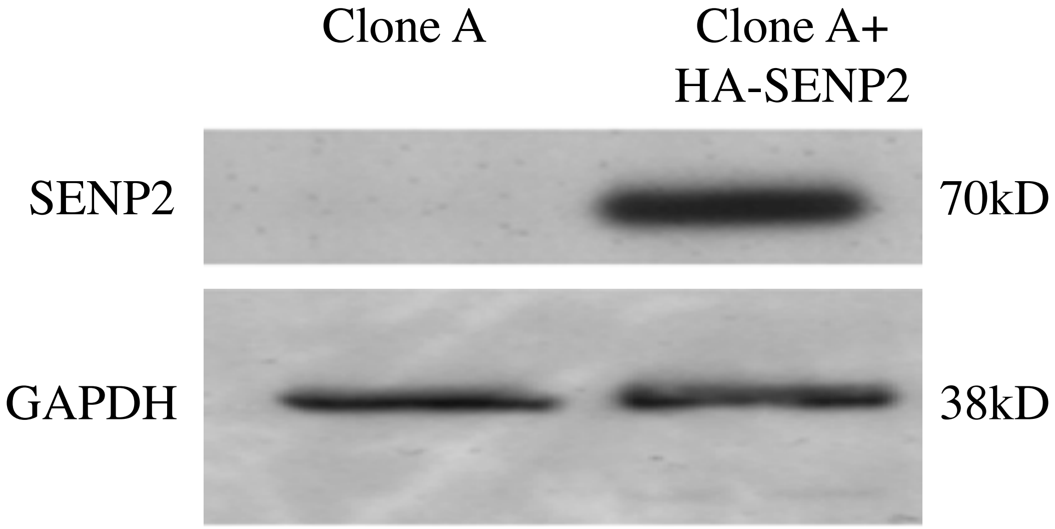

Supplementary Figure 6: Full length western blot of Figure 3 A

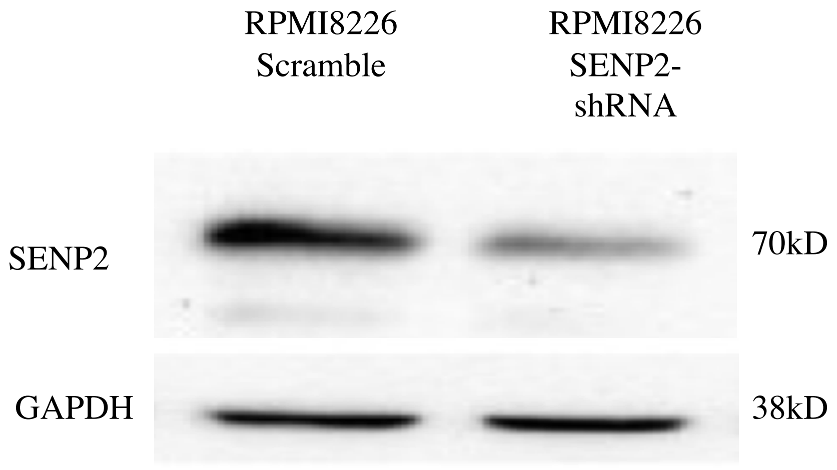

Supplementary Figure 7: Full length western blot of Figure 4 A

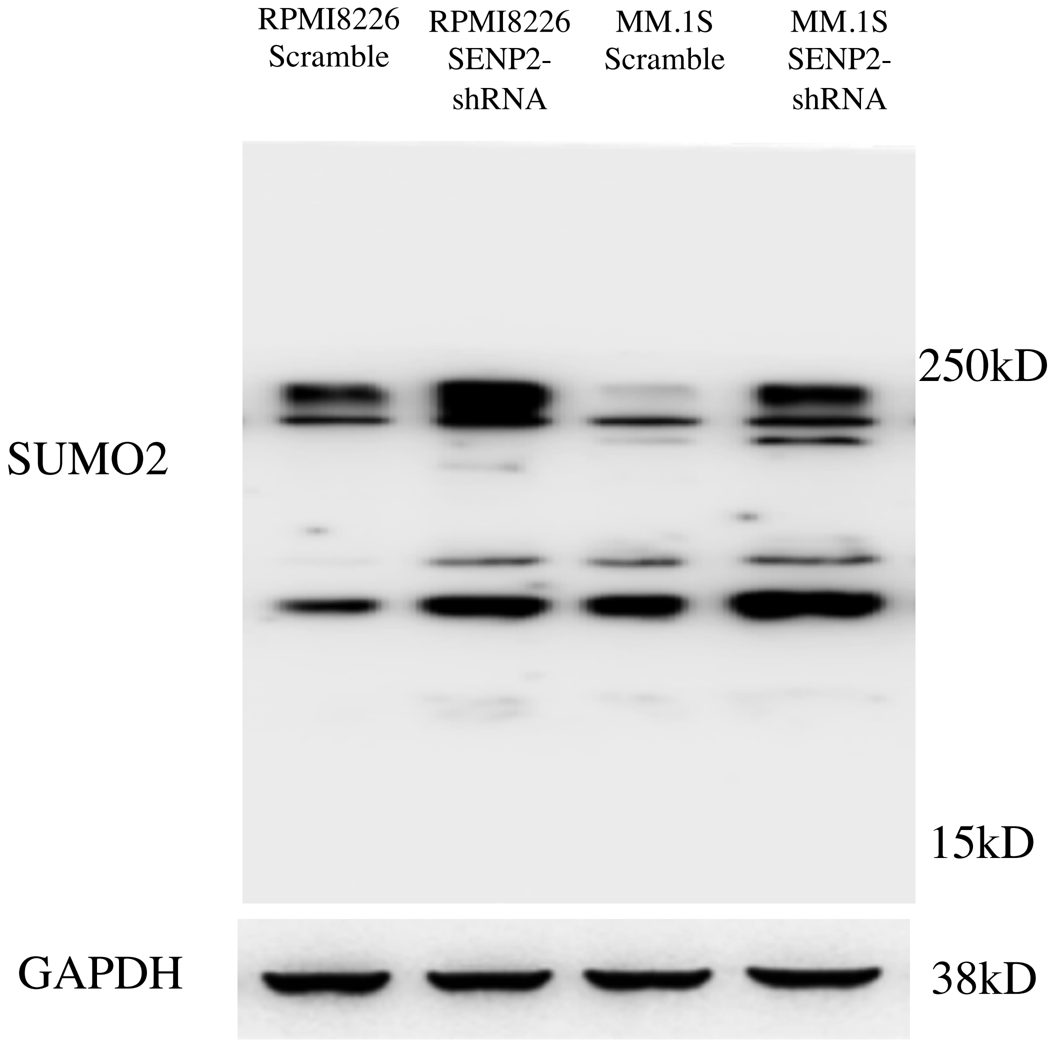

Supplementary Figure 8: Full length western blot of Figure 4 B

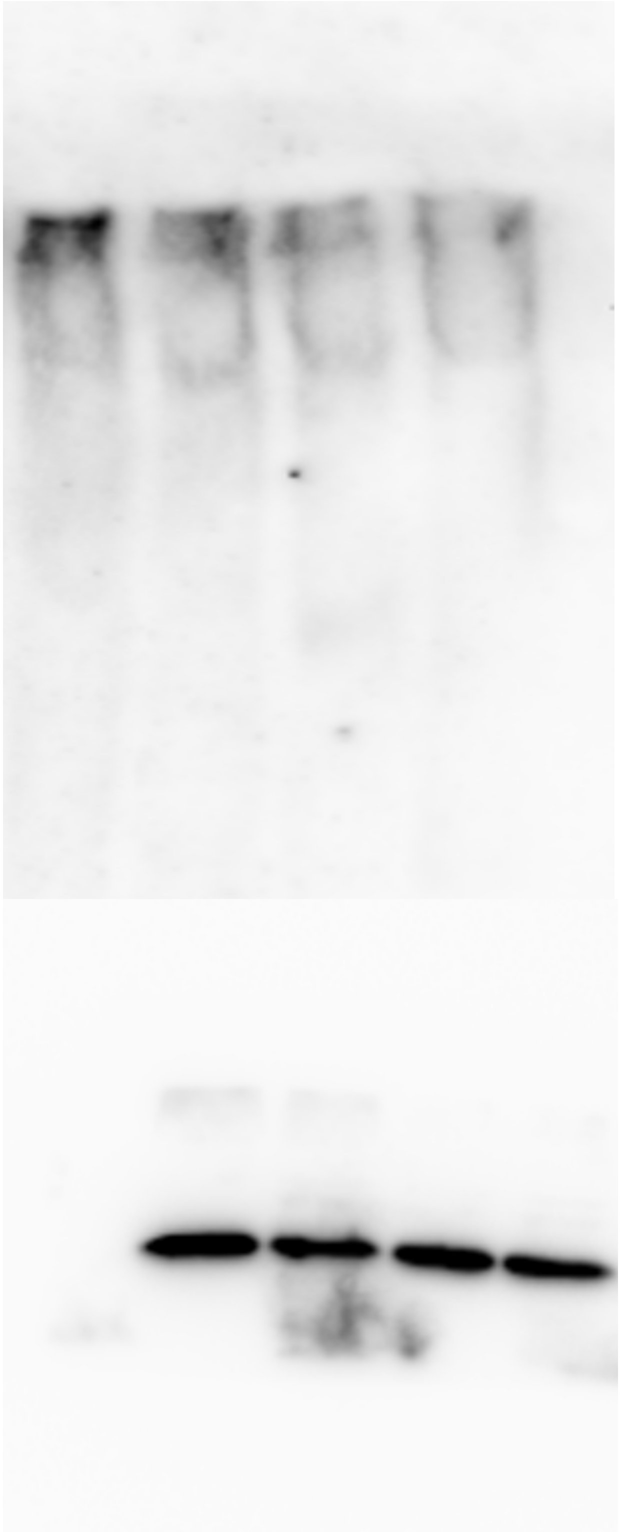

Supplementary Figure 9: Full length western blot of Figure 4 C

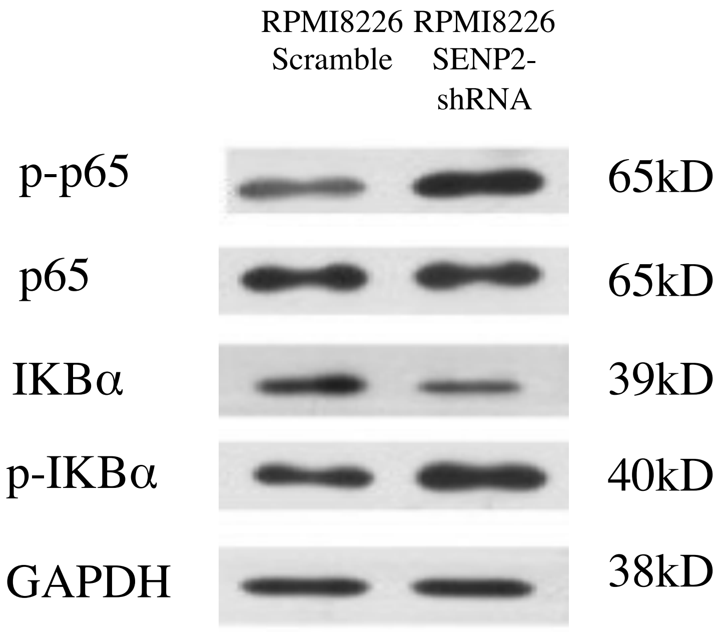

Supplementary Figure 10: Full length western blot of Figure 4 E

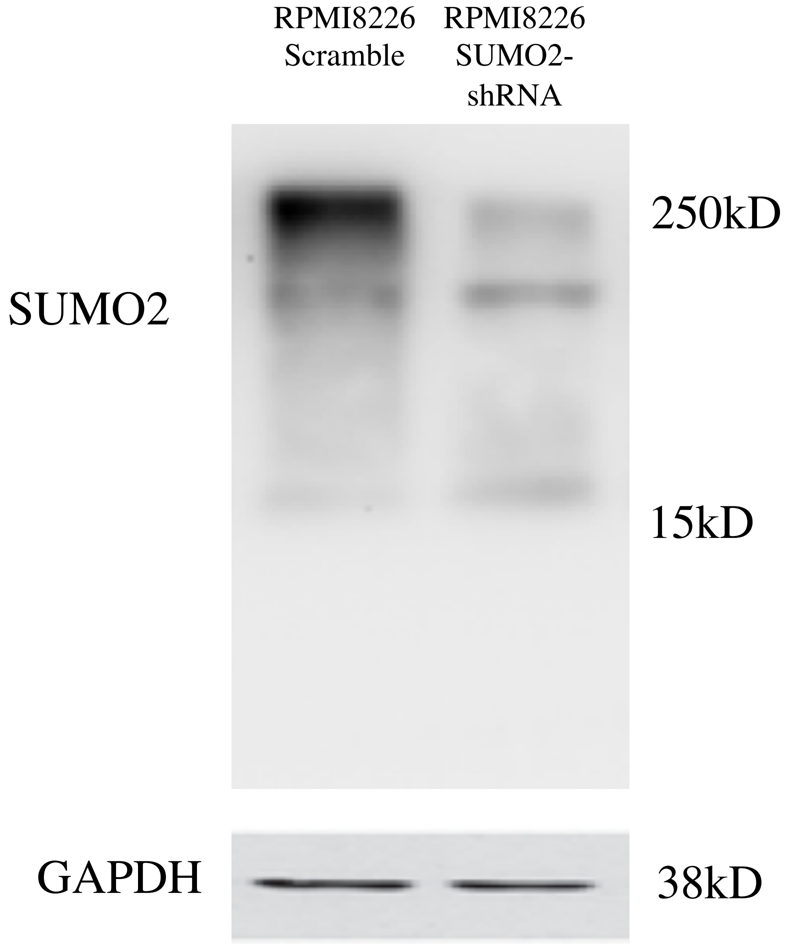

c

Supplementary Figure 11: Full length western blot of Figure 4 F

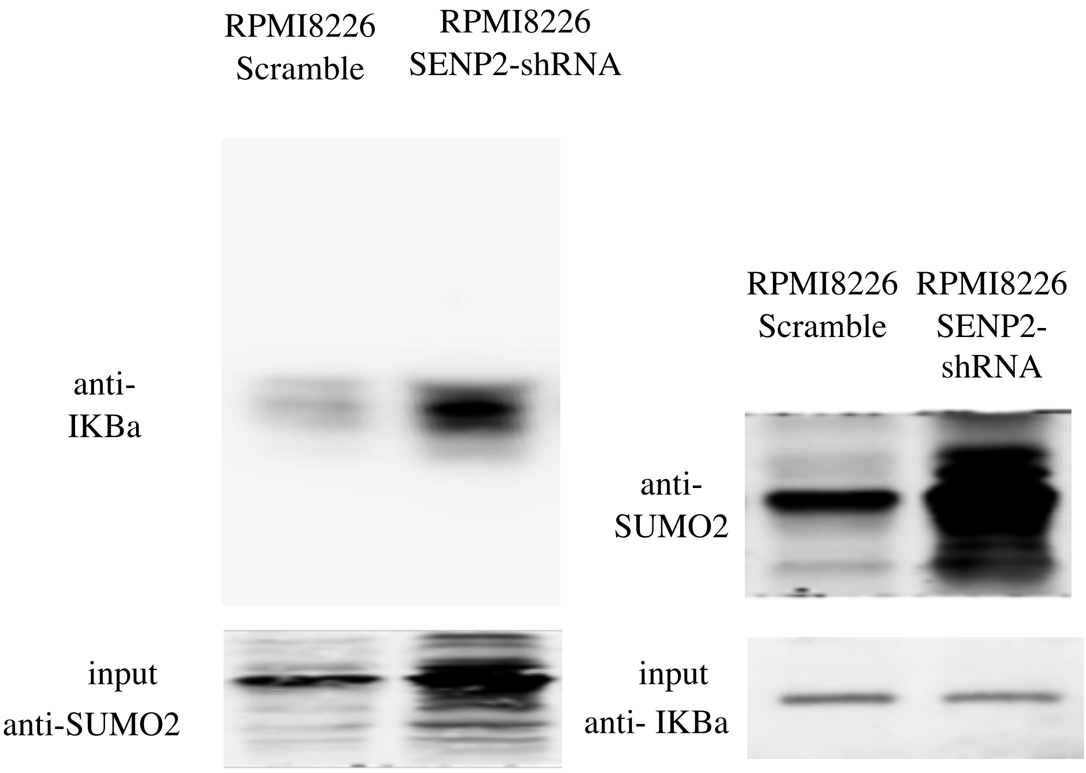

Supplementary Figure 12: Full length western blot of Figure 4 H
